# A New Filler for Epoxy Resin: Study on the Properties of Graphite Carbon Nitride (g-C_3_N_4_) Reinforced Epoxy Resin Composites

**DOI:** 10.3390/polym12010076

**Published:** 2020-01-02

**Authors:** Tingting Wang, Bo Song, Li Wang

**Affiliations:** 1 School of Mechanical & Electrical and Information Engineering, Shandong University, Weihai 264209, China; wt19900923@163.com; 2 Marine College, Shandong University, Weihai 264209, China

**Keywords:** g-C_3_N_4_, epoxy nanocomposites, mechanical properties, thermal properties, particle-reinforcement

## Abstract

In this study, graphitic carbon nitride (g-C_3_N_4_) as a novel filler was used for fabricating epoxy nanocomposites. The static mechanical, dynamic thermal-mechanical properties and thermostability of as-prepared g-C_3_N_4_/epoxy nanocomposites were significantly ameliorated compared with that of the pure epoxy matrix. The tensile modulus and flexural modulus of g-C_3_N_4_/epoxy nanocomposites increased by 31.81% and 28.28%, respectively. Meanwhile, the tensile and flexural strength was also improved by 16.02% and 12.67%, respectively. The g-C_3_N_4_/epoxy nanocomposites exhibited an increased storage modulus and glass transition temperature. The markedly improved mechanical and viscoelasticity properties were attributed to the stronger interfacial interaction caused by enlarged contact area and increased chemical bonding, and enhanced mechanical interlocking on the interface. The loss factor of epoxy nanocomposites also raised by 40% due to the comprehensive effect of frication caused by the relative slip between nanosheets, micro-constrained layer damping structure and the reversible cycle of breakage and re-established of the hydrogen bond. Meanwhile, the 10% weightlessness temperature (T*_initial_*), semi weightlessness temperature (T*_half_*) of g-C_3_N_4_/epoxy nanocomposites have increased by about 15 °C and 14 °C, respectively.

## 1. Introduction

Epoxy resin as an excellent matrix material was used widely in the field of aerospace, automobile and architecture. However, some properties of epoxy resin are far from satisfactory at present [1,2,3,4]. Therefore, various fillers such as graphene [5], carbon nanotubes [6], clay minerals [7,8] and polymeric particles [9] were used to ameliorate epoxy matrix. Among them, the two-dimensional (2D) nanosheets as a novel nanomaterial have attracted tremendous attention. Due to the ultimate two-dimensional anisotropy, nanoscale thickness and other unique properties [10,11,12,13], the two-dimensional (2D) nanosheets have exhibited excellent application potential in the areas of electronic [14], sensors [15], energy storage [16] and polymer reinforcement. A typical approach to fabricate nanocomposites reinforced with two-dimensional (2D) nanosheets is to employ intercalation chemistry of layered materials such as graphite, clay minerals and layered double hydroxides, etc. [7]. The clay minerals as an outstanding 2D materials have been widely used for the enhanced mechanical properties of materials due to their uniquely layered structure [17,18,19,20]. Similarly, graphene also has attracted the focus of the scientific community since the advent in 2004 [21] because of the unique structure and excellent properties. A great number of studies on the enhanced physical properties and improved functionalities of graphene have also been conducted and showed significant improvement [22,23,24,25].

Recently, graphitic carbon nitride (g-C_3_N_4_) as a polymeric semiconductor with graphite-like structure has attracted extensive attention of researchers all over the world owing to its low-cost and good chemical stability. C_3_N_4_ is a carbon-nitrogen compound that has been discovered [26] and concerned for a long time. It has several kinds of allotropes, among which graphite-like carbon nitride (g-C_3_N_4_) is the most stable one at room temperature and atmospheric pressure. Allotropes of other structural forms are stable only at high temperatures, which are hard to prepare and difficult to study. Wang et. al. [27] proposed that g-C_3_N_4_ could be used as a metal-free photocatalyst to decompose water to produce hydrogen under visible light, which has triggered an upsurge in the research of carbon nitride in the field of photocatalysis. The 2D monolayer of g-C_3_N_4_ exhibited a considerable specific surface area and abundant residual functional groups (−NH_2_ or −NH). These groups can act as active sites for the formation of strong interface bonding or grafting other functional groups to improve its compatibility with polymer matrices. All these characteristics confirmed that the enormous potential of g-C_3_N_4_ as an ideal reinforcement for the high-performance composites with excellent mechanical properties. Shi et al. [28] investigated the influence of g-C_3_N_4_ nanosheets on thermal stability and mechanical properties of sodium alginate films. The initial thermal degradation temperature increased by 29.1 °C and the tensile modulus remarkably increased from 60 to 3540 MPa after adding 6 wt.% g-C_3_N_4_ into the matrix. Furthermore, Shi et al. [29] studied the thermal and mechanical properties of g-C_3_N_4_/polypropylene nanocomposite in the following research. The initial and half thermal degradation temperature was raised by 14.6 °C and 27.7 °C, respectively. The storage modulus was also increased from 2445 MPa to 2783 MPa. Sareshkeh et al. [30] focused on the mechanical and thermal properties of g-C_3_N_4_ reinforced epoxy-vinyl resin. The presence of g-C_3_N_4_ increased to the tensile strength and Izod-impact strength by 49.2% and 25.37%, respectively, and promoted the T*_g_* (glass transition temperature) and IDT (initial decomposition temperature) by 6.46% and 12.05%, respectively. The interlaminar shear strength and interfacial shear strength of composite laminates were increased from 51.84 to 72.09 MPa and 44.62 to 73.41 MPa, respectively. Although the remarkable progress in g-C_3_N_4_ reinforced composites have been achieved, the potential of g-C_3_N_4_ in the design and fabrication of high-performance and functional composites still remain to be further explored and utilized.

Motivated by the above discussion, epoxy nanocomposites reinforced with g-C_3_N_4_ was prepared. The static mechanical and dynamic thermomechanical properties of g-C_3_N_4_/epoxy nanocomposites were investigated. The thermogravimetric performance was also studied. As far as we know, there is no relevant research on g-C_3_N_4_ reinforced epoxy nanocomposites.

## 2. Materials and Methods

### 2.1. Materials

Urea (A. R.) was purchased from Aladdin (Shanghai, China). Epoxy resin (HT-723A) and curing agent (HT-723B) were supplied by Wells Advanced Materials Co., Ltd. (Shanghai, China).

### 2.2. Preparation of g-C_3_N_4_

**Figure 1:**
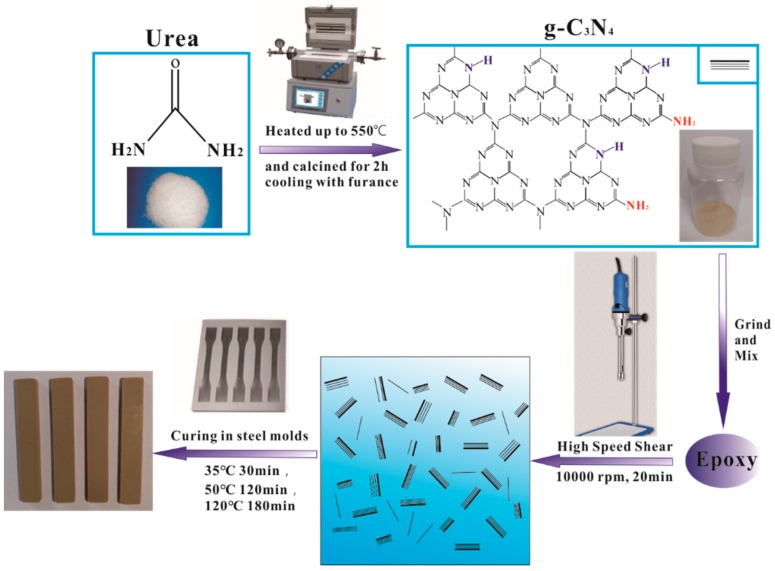
Schematic representation of synthesis of g-C_3_N_4_ using urea as a precursor and fabrication of g-C_3_N_4_/epoxy nanocomposites.

The preparation process of g-C_3_N_4_ and epoxy nanocomposites is shown in Figure 1. The g-C_3_N_4_ were prepared through the thermal decomposition of urea. In a typical synthesis process (Figure 1), a proper amount of urea power was placed into a 100 mL ceramic crucible with lid. The crucible was heated at 550 °C for 2 h (heated rate: 20 °C/min) in a tubular furnace and then cooled naturally with furnace. The as-prepared g-C_3_N_4_ was ground into fine powder for the subsequent procedure.

### 2.3. Preparation of Epoxy Nanocomposites

The preparation of epoxy nanocomposites is also shown in Figure 1. A certain proportion of g-C_3_N_4_ powder (0.0 wt.%, 0.5 wt.%, 1.0 wt.%, 1.5 wt.%, 3.0 wt.%, 4.0 wt.% and 5.0 wt.%) was added into epoxy resin and mixed by a high-speed shear dispersion homogenizer at 10,000 rpm for 20 min, and then the curing agent was mixed into the above suspension at a mass ratio of 30:100. The mixture was casted into a steel mold for curing after defoaming treatment. A typical curing procedure was as follows: 35 °C for 30 min, 50 °C for 120 min and then 120 °C for 180 min. According to the content of g-C_3_N_4_ in an epoxy matrix, the as-prepared g-C_3_N_4_/epoxy nanocomposites were labeled as EP-CN0.0/0.5/1.0/1.5/3.0/4.0/5.0, respectively.

### 2.4. Characterizations and Measurements

The crystal structure of g-C_3_N_4_, epoxy and nanocomposites were characterized by X-ray diffraction (XRD, Ultima IV with Cu-Kα radiation, Rigaku D/max-rB, Tokyo, Japan). The surface functional groups of g-C_3_N_4_ were investigated by flourier transform infrared spectroscopy (FTIR, VERTEX70, Bruker, Mannheim, Germany). The morphologies of g-C_3_N_4_ was observed using transmission electron microscopy (TEM, JEOL 2010, JEOL, Tokyo, Japan) and scanning electron microscope (SEM, Nova NanoSEM450, FEI, Hillsboro, OR, USA). The specific surface area (SSA) and pore size distribution of g-C_3_N_4_ were tested by nitrogen physisorption apparatus (Nova Station C, Quantachorme, Boynton Beach, FL, USA).

The static mechanical properties (tensile and flexural) of g-C_3_N_4_/epoxy nanocomposite was measured using a universal testing machine (Exceed 45, MTS, Shenzhen, China) with a high-precision load cell with self-identification function (TEDS). According to the tensile standard of ASTM D638, a dumbbell-shaped specimen was selected, with a total length of 165 mm, a width of 13 mm at the middle, 19 mm at both ends, a gauge length of 50 mm and a thickness of 3.2 mm. The standard of ASTM D790 was applied for the flexural test. At least five valid data were obtained from seven samples in each group. The fracture surfaces of the tensile test samples were investigated by SEM.

Dynamic thermal mechanical behavior was investigated by a dynamic thermomechanical analyzer (DMA Q800, TA, New Castle, PA, USA) at a fixed frequency of 1 Hz and a temperature range from 25 to 180 °C with a heating rate of 3 °C/min. Three points bending mode was chosen and the samples were machined with dimensions of 60 mm × 10 mm. The linear viscoelastic region was probed by amplitude scanning and the amplitude of 15 μm was determined. The loss factor was also measured by DMA at the mode of temperature step multi frequency scanning in a temperature range of 25–35 °C with multiple excitation frequencies. Considering to expand the test range as much as possible without resonance, these frequencies, 1/5/10/15/20/25/30 Hz, were chosen.

Thermogravimetry (TG) analysis of nanocomposites was carried out on a DSC/TGA simultaneous thermal analyzer (DSC1, METTLER TOLEDO, Zurich, Switzerland) from 50 to 750 °C at a heating rate of 10 °C/min.

## 3. Results and Discussion

### 3.1. Morphology and Structure Characterization of g-C_3_N_4_

**Figure 2:**
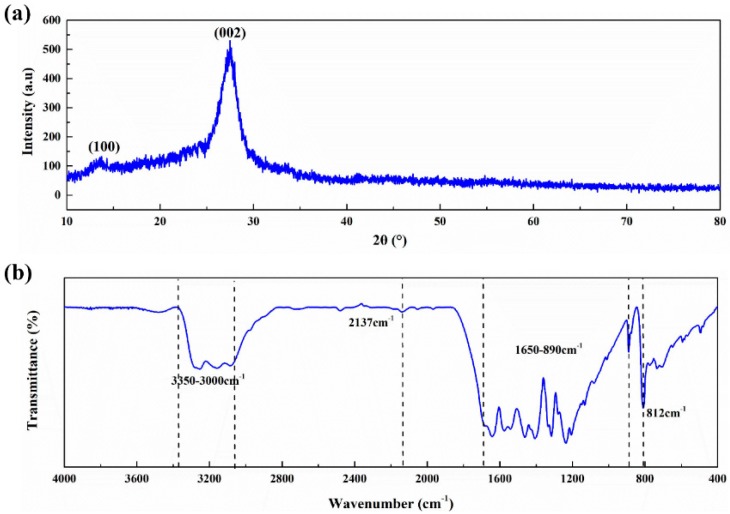
Spectra of g-C_3_N_4_: (**a**) X-ray Diffraction (XRD); (**b**) Fourier Transform Infrared Spectrometer (FT-IR).

Two typical diffraction peaks can be observed in the XRD spectra of g-C_3_N_4_, as shown in Figure 2a. The diffraction peak at 13.38° is attributed to in-plane (100) ordering of tri-s-triazine units with an interlayer distance of 0.670 nm, while the diffraction peak at 27.54° normally corresponds to the interlayer stacking of conjugated aromatic structures (002). The peak of (002) can be indexed to the graphitic materials, which corresponds well to the interlayer distance (0.325 nm) of g-C_3_N_4_ [31,32].

The FTIR analysis of g-C_3_N_4_ is shown in Figure 2b. The formation of g-C_3_N_4_ with the tri-s-triazine building units is confirmed. The absorption peak at 812 cm^−1^ is attributed to the out-of-plane bending vibration characteristic of heptazine rings and the characteristic mode of the triazine units of g-C_3_N_4_ [33]. The weak peak at 2137 cm^−1^ is assigned to the C≡N triple bonds [34]. Due to the incomplete condensation, there are more or less undeleted amino groups in g-C_3_N_4_ prepared by thermal condensation polymerization. The absorption peaks in the range of 1650–890 cm^−1^ represent the typical stretching vibration modes of heptazine-derived repeating units and connected units of C−N(−C)−C (full condensation) or C−NH−C (partial condensation) [35]. The broad band in the range of 3500–3000 cm^−1^ is attributed to the stretching mode of the O−H bond and the vibration mode of the N−H bond [36].

**Figure 3:**
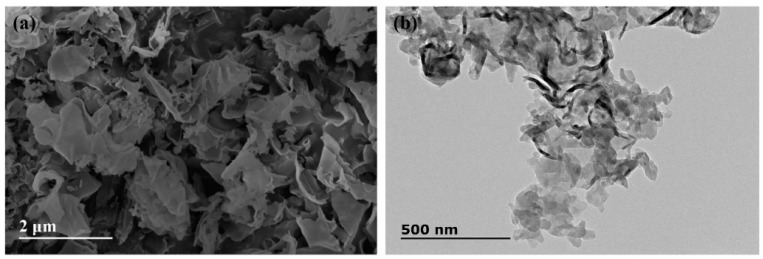
Micrograph of g-C_3_N_4_: (**a**) scanning electron microscope (SEM); (**b**) transmission electron microscopy (TEM).

The microscopic morphology of as-obtained g-C_3_N_4_ powder was investigated by TEM and SEM, as shown in Figure 3. It is clear that the power is composed of numerous two-dimension sheet structures with irregular edge and slight curls (Figure 3a). The ultrafine nanosheets with several tens of nanometers in size are observed by TEM (Figure 3b). The exhibited morphology demonstrates that the pure phase and ultrathin g-C_3_N_4_ nanosheets were obtained in our experiment.

**Figure 4:**
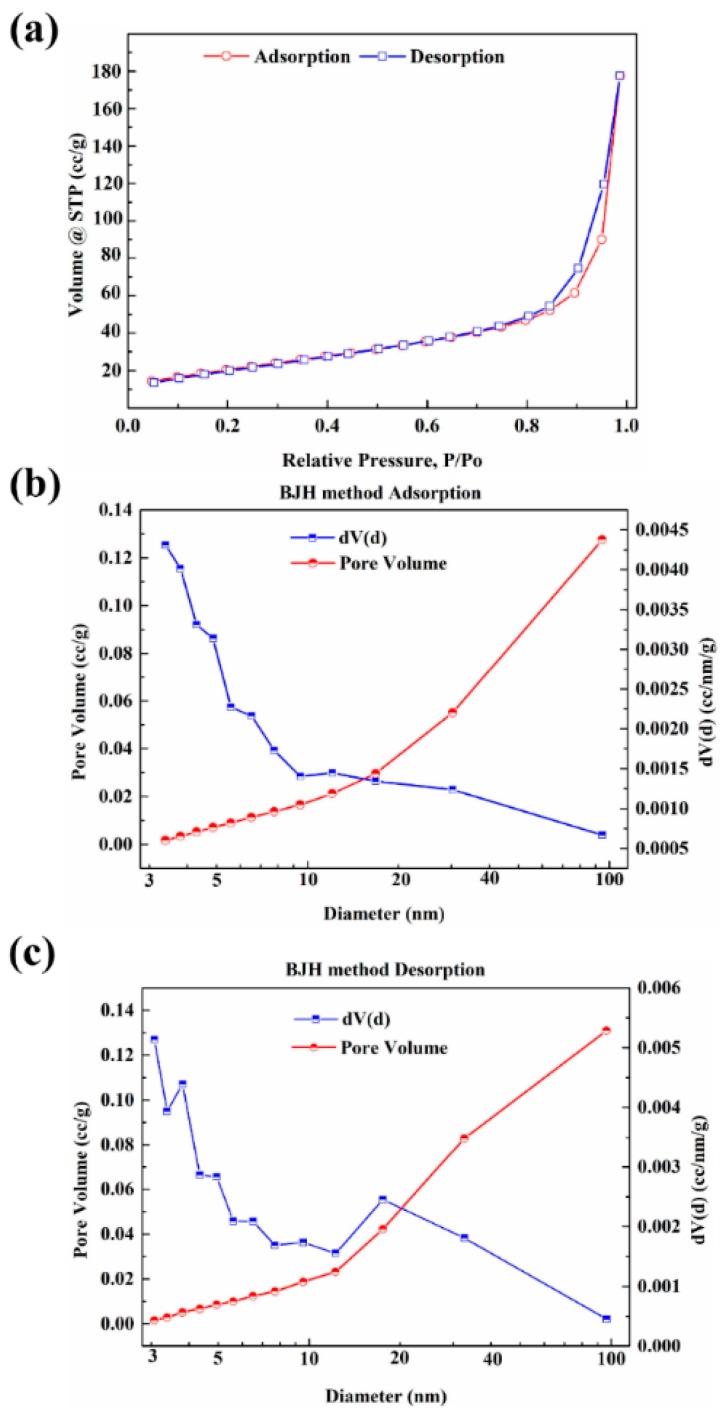
The N_2_ adsorption and desorption isotherms (**a**) and pore size distribution and pore volume of g-C_3_N_4_; (**b**) adsorption; (**c**) desorption.

The ideal g-C_3_N_4_ is a perfect planar lattice structure formed only by the sp^2^ hybridization of C and N atoms. However, a certain degree of curling deformation may occur in the plane of g-C_3_N_4_ due to the repulsion between N atoms [37] and stress release. Moreover, there are numerous mesopores of several tens of nanometers in size on the nanosheet, which is related to the precursors used in the preparation [38,39]. The intermediate products of g-C_3_N_4_ were synthesized with the pyrolysis of urea and a large amount of ammonia and steam were released, which contributed to the expansion of the stacked-layer and promoted the graphitization of g-C_3_N_4_, thus making the thickness of the single-layer thinner and promoting the formation of nano-lamellar porous morphology. The N_2_ adsorption isotherms and pore size distribution of g-C_3_N_4_ are shown in Figure 4. The specific surface area and pore size distribution were calculated by multi-point BET and BJH method, respectively. The results show that the g-C_3_N_4_ has a considerable specific surface area (97 m^2^/g) and pore volume (0.45 cm^3^/g). In addition, the pore size and distribution analysis confirm that micropores and mesopores are both existed on the g-C_3_N_4_ surface and micropores have a dominant position on pore structure of g-C_3_N_4_.

### 3.2. Dispersion of g-C_3_N_4_

**Figure 5:**
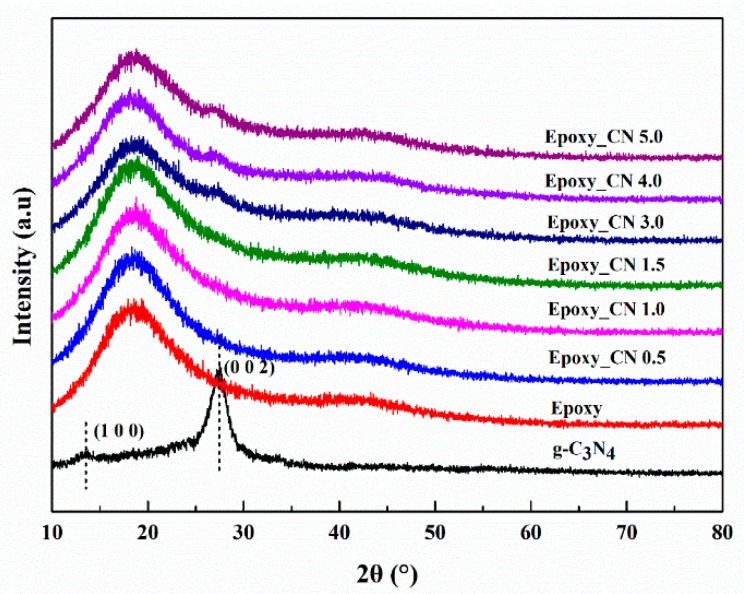
X-ray Diffraction (XRD) patterns of the epoxy and g-C_3_N_4_/epoxy nanocomposites.

**Figure 6:**
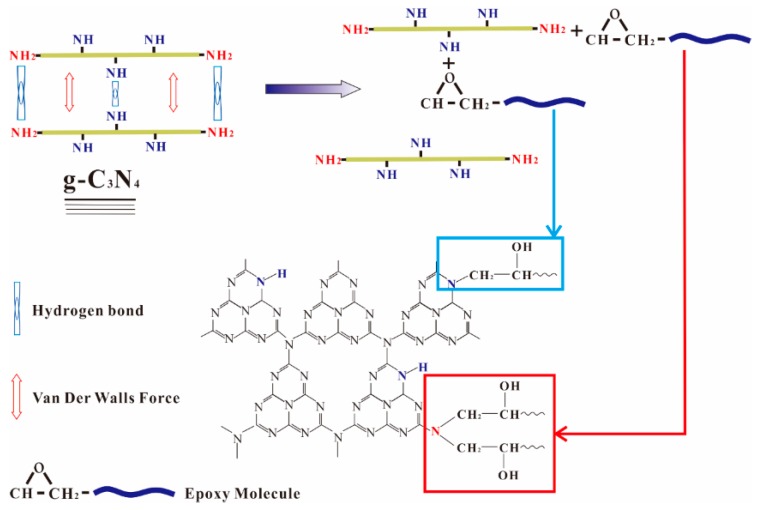
Schematic representation of the interaction between g-C_3_N_4_ and the epoxy resin.

The state of fillers in the cured matrix greatly affects the properties of the nanocomposites. The XRD technique is a crucial method for determining the exfoliation degree of graphene in the studies of graphene reinforced polymer composites and the disappearance of stack peaks always indicates a uniformly dispersion [5]. The XRD patterns of epoxy and g-C_3_N_4_/epoxy nanocomposites are shown in Figure 5. Here, a characterized peak of g-C_3_N_4_ centered at 26.6° appears in the XRD spectra of epoxy nanocomposites when g-C_3_N_4_ loading is no less than 3 wt.% which indicates that the larger loading of g-C_3_N_4_ (≥3 wt.%) led to incomplete exfoliation in our experiments. Compared with graphene, the dispersion of g-C_3_N_4_ in epoxy resin has certain advantages in terms of surface functional groups. The layers of g-C_3_N_4_ are bound by a hydrogen bond and Van der Waals force. In the dispersion process, epoxy resin permeated into the interlayer and the nanosheets were fully encapsulated by epoxy under shear stress. As shown in Figure 6, the residual functional groups (−NH_2_ or −NH) on g-C_3_N_4_ can react with the epoxy groups of the matrices, which impels the intercalation of epoxy into the interlamination and then promotes the dispersion of g-C_3_N_4_ in epoxy matrix.

### 3.3. Static Mechanical Properties

**Figure 7:**
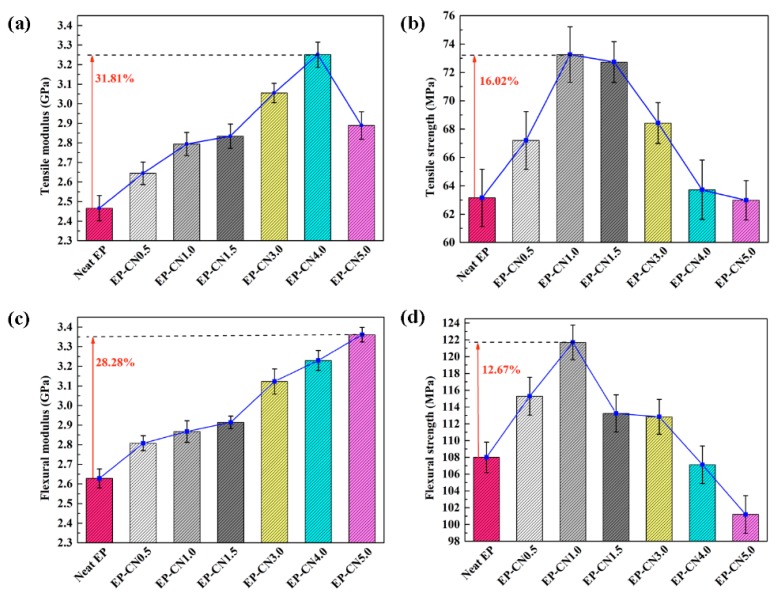
Tensile and flexural properties of neat epoxy and g-C_3_N_4_/epoxy nanocomposites with different filler loadings: (**a**) tensile modulus; (**b**) tensile strength; (**c**) flexural modulus; (**d**) flexural strength.

The tensile and flexural properties of pure epoxy and g-C_3_N_4_/epoxy nanocomposites are depicted in Figure 7. Compared to pure epoxy, the tensile modulus of nanocomposites is observably enhanced by adding g-C_3_N_4_. The maximum growth of the tensile modulus (from 2466 ± 64 to 3250 ± 64 MPa, 31.81%) was obtained from EP-CN4.0. Meanwhile, the flexural modulus of g-C_3_N_4_/epoxy nanocomposites also exhibits an increasing trend (raised from 2628 ± 48 to 3361 ± 37 MPa) with the increased loading of g-C_3_N_4_. EP-CN5.0 achieves the strongest value of flexural modulus (3361 ± 37 MPa) with an increased rate of 28.28%. The markedly enhanced tensile and flexural modulus of nanocomposites can be attributed to the good interfacial bonding between g-C_3_N_4_ nanosheets and epoxy. As displayed in Figure 6, the residual functional groups (−NH_2_ or −NH) on g-C_3_N_4_ contributes to the formation of stronger interfacial interaction between g-C_3_N_4_ and epoxy. In addition, the curly structure and the large specific surface area of the g-C_3_N_4_ nanosheet also enhance the mechanical interlocking effect with the matrix.

**Figure 8:**
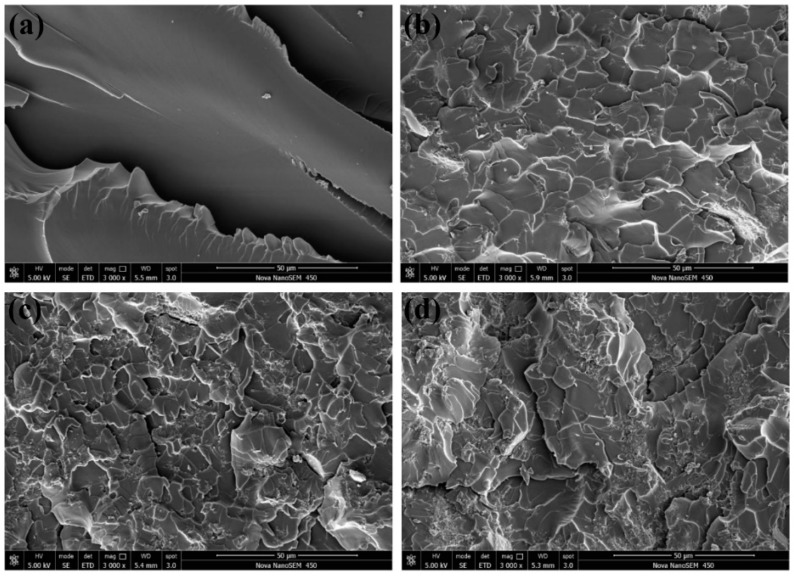
Fracture surface of neat epoxy (**a**), EP-CN1.0 (**b**), EP-CN3.0 (**c**) and EP-CN5.0 (**d**).

**Figure 9:**
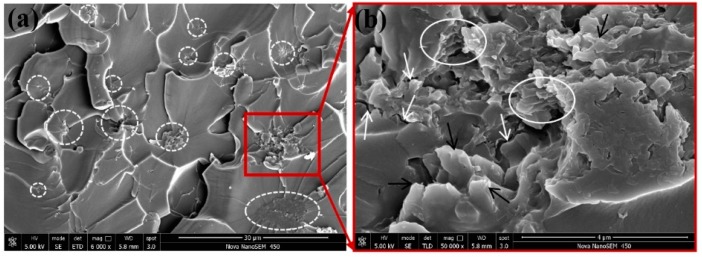
(**a**) Distribution and interface quality of g-C_3_N_4_ in the matrix of EP-CN3.0; (**b**) The interface quality of g-C_3_N_4_ in the matrix of EP-CN3.0 in high resolution.

The increasing of the tensile strength (16.02%) and flexural strength (12.67%) of nanocomposites are not obvious enough as a modulus, and a considerable number of particle enhancement nanocomposites presented similar results. The fracture morphology of epoxy nanocomposites after the tensile test was investigated using SEM. The fracture section of epoxy is a relatively smooth surface with wave morphology (Figure 8a) while the nanocomposites exhibit rougher fracture morphology. As shown in Figure 8b, these rough surfaces consist of numerous small faceted features. The formation of homogeneous morphology is ascribed to the physical barrier effect of g-C_3_N_4_ originated from the good interfacial adhesion between g-C_3_N_4_ and the epoxy matrix [40]. The direction of the crack propagation was forced to change when encountering the stiff g-C_3_N_4_, thus resulting in more consumption of the energy and toughening of the matrix. The quantity of deviated cracks depends on the number of particles the crack encountered and this point can be confirmed by the refinement of the surface grain reflected in Figure 8b,c. At low loading, g-C_3_N_4_ nanosheets existed in a state of island-like with good interface condition in the matrix. The agglomerates appeared with further increasing of g-C_3_N_4_ and the agglomeration and homogeneous distribution of g-C_3_N_4_ were coexistent in the matrix of EP-CN3.0 and the “islands” are marked by the white dotted circle in Figure 9a, which also confirm the previous analysis of the XRD pattern. The more detailed observation of the agglomerate is presented in Figure 9b. The surface of g-C_3_N_4_ is completely infiltrated by epoxy and the laminated structure of g-C_3_N_4_ sheets are clearly exhibited on the surface. Although there are some gaps (marked by white arrows), the agglomerate is still completely infiltrated by the matrix, exhibiting good bonding with the matrix (marked by black arrows). Nevertheless, as the increased loading promotes the formation of more strong interfacial interaction, it also leads to poor dispersion of the g-C_3_N_4_. The existence of excessive g-C_3_N_4_ results in the formation of large-size agglomerates and the reduction of crack deviation efficiency. The agglomerates with layered structure dramatically reduce the specific surface area and wettability of g-C_3_N_4_ thus the mechanical properties of nanocomposites decrease. On the whole, the enhancement of g-C_3_N_4_ is comparable to that of graphene [5], but a larger loading of g-C_3_N_4_ would be required to achieve the same enhancement effect.

### 3.4. Dynamic Thermal Mechanical Properties

**Figure 10:**
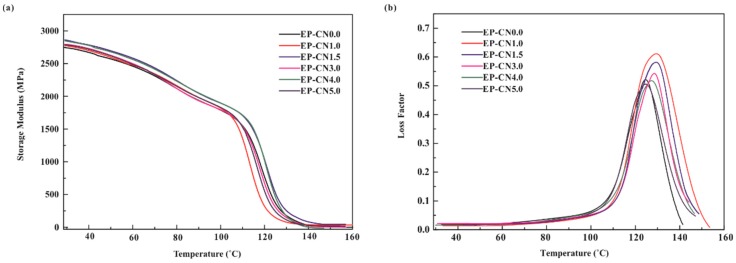
Dynamic mechanical properties of neat epoxy and g-C_3_N_4_/epoxy nanocomposites (EP-CN1.0, EP-CN3.0, EP-CN5.0): (**a**) storage modulus; (**b**) loss factor.

**Figure 11:**
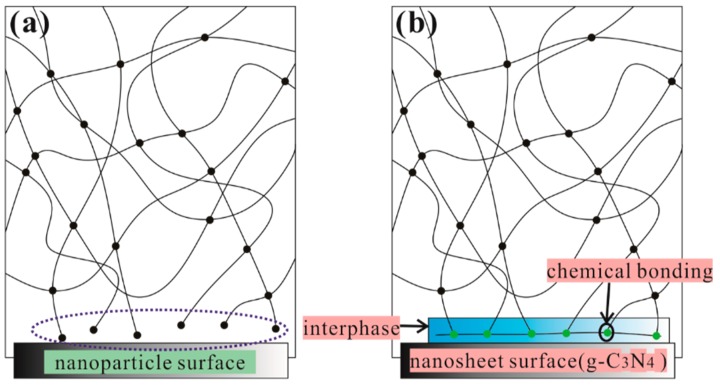
Schematic Representation: (**a**) the disruption of the matrix network near the surface; (**b**) the retarding of the mobility of the epoxy molecular chain due to strong attractive interactions.

The storage modulus plots for pure epoxy and g-C_3_N_4_/epoxy nanocomposites are shown in Figure 10a. One can recognize that the g-C_3_N_4_/epoxy nanocomposites exhibit an increased storage modulus at the glassy state region due to the reinforcing effect of the g-C_3_N_4_ nanosheets, compared to pure epoxy. The storage modulus of EP-CN5.0 is 2892 MPa at 30 °C 1 Hz while that of epoxy is 2754 MPa. The effect of loading on the enhancement is similar to that of static bending test, but the storage modulus of nanocomposites decreases significantly with the increase of temperature, which is mainly determined by the matrix material used in our experiment [41]. Figure 10b shows the loss factor plot for neat epoxy and epoxy composites containing g-C_3_N_4_. T*_g_* is associated with the fundamental changes in polymer chain dynamics, thermal stability and many other critical applications [42,43]. Generally, the presence of the nanoparticle has both positive and negative effects on T*_g_* [44]. Nano-scale particles would hinder the movement of matrix molecular matrix chains, but can also limit the number of functional groups in the local vicinity; thus, leading to the disproportionality of the local functional groups between the nanoparticles and the matrix and the disruption of the matrix network near the surface of the nanoparticles (Figure 11a). The T*_g_* of EP-CN1.0 and EP-CN1.5 both increases about 8 °C in comparison to that of the epoxy matrix. It is also worth noting that when the loading is more than 3 wt.%, as the loading increases, the enhancing effect on T*_g_* weakens. It can be seen from Figure 10b that, in addition to EP-CN5.0, the T*_g_* of the other several kinds of addition configuration have all been improved to varying degrees which is attributed to the restriction effect of strong attractive interactions formed by the reaction between residual functional groups (−NH_2_ or −NH) of g-C_3_N_4_ and epoxy group on the motion of epoxy molecular chain. In addition, the functional groups carried by g-C_3_N_4_ remedy the disproportionality of local functional group and played a role in repairing local grids, as shown in Figure 11b. The restrictive effect of the mechanical interlocking originated from the curling structure of nanosheets on the movement of the polymer matrix molecule chain cannot be neglected either. The geometric constraints of the nanocomposites on the mobility of the molecules of the polymer were also verified in carbon-nanoparticles (CNTs, GO et al.) reinforced epoxy composites by simulation and experiment [45,46]. However, the T*_g_* value of EP-CN5.0 with an excessive amount of g-C_3_N_4_ is 3 °C below that of pure epoxy due to the deterioration of dispersion, reduction of interfacial area and formation of fillers-rich or poor regions [2,47].

**Figure 12:**
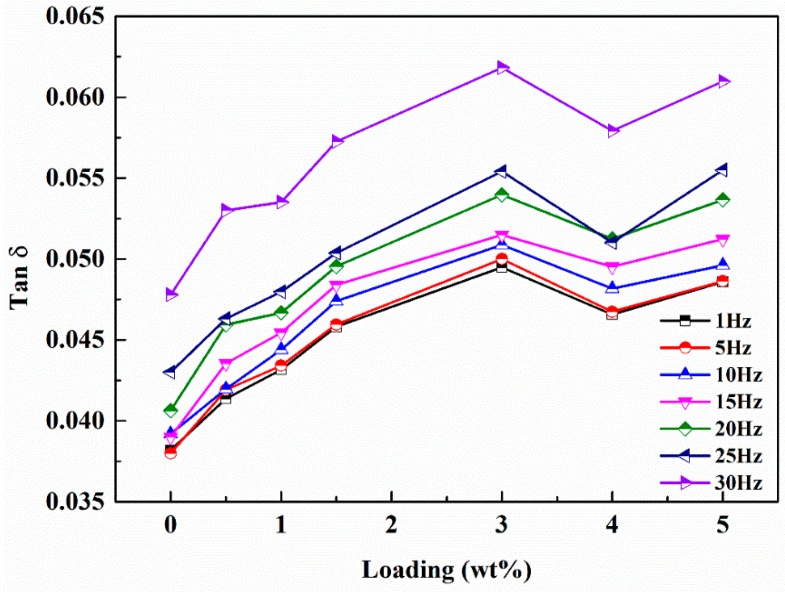
Loss factor of neat epoxy and g-C_3_N_4_/epoxy nanocomposites with different frequencies at 30 °C by the method of temperature step multi-frequency scanning.

**Figure 13:**
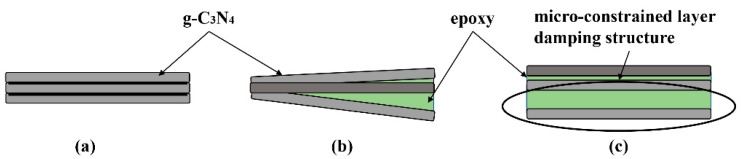
The schematic diagram of the possible existence form of stacked structures in the matrix in the state of insufficiently dispersed state: (**a**) the original structure had not been changed at all; (**b**) the interlayer interface had been partially separated; (**c**) the interlayer interface had been completely separated, but the stacking structure is still maintained.

The loss factor characterizes the damping properties of materials. In our experiments, the loss factors were also investigated in the mode of step temperature multi-frequency scanning and the influence of temperature on loss factor can be neglected in the test range. As shown in Figure 12, the loss factors of the nanocomposites increase significantly with the increased g-C_3_N_4_ loading and the maximum value is obtained at 3 wt.% loading. The loss factor of EP-CN3.0 under the excitation frequency of 1 Hz at 30 °C increased from 0.035 to 0.049. It is generally believed that the energy dissipation originated from interfacial friction is the main reason for the enhancement of damping properties of nanocomposites. However, the strong interfacial interaction between g-C_3_N_4_ and matrix means the critical interfacial shear strength could be relatively high and the interfacial slip is not easy to occur. Nevertheless, the loss factor of nanocomposites has been significantly improved, which indicates the existence of other forms of energy dissipation. Moreover, it is possible that the increased loss factors are related to the multiscale laminated structure of g-C_3_N_4_. The multiscale nanoparticles exhibited both fully dispersed and insufficiently dispersed states in the matrix. In the state of fully dispersed, most of the particles existed in the form of non-oriented single nanosheet, while in the state of insufficiently dispersed the existence of stacked structures in the matrix can be divided into three situations, as shown in Figure 13: (a) the original structure had not been changed at all; (b) the interlayer interface had been partially separated; (c) the interlayer interface had been completely separated, but the stacking structure is still maintained. In the case of b and c, the micro-constrained layer damping structure was formed by epoxy matrix entered into the separated layers and energy dissipation was realized by shear deformation of the matrix entering the interlayer. In situation a, the friction caused by the relative slip between nanosheets is the principal source of energy dissipation. The study of Shi [28] indicated that there were a large number of hydrogen bonds between layers of g-C_3_N_4_. Therefore, in addition to interfacial friction, the reversible cycle of breakage and re-established of hydrogen bond (Figure 5) between layers could also generate energy dissipation. On the whole, we believed that the damping enhancement of g-C_3_N_4_ reinforced epoxy nanocomposites may be the result of a combination of above energy dissipation modes. Another point worth noting is the peculiar change with a loading of 5 wt.% which might be related to the gradual deterioration of the interface between agglomerates and matrix. As the dimension of the agglomerates became larger, they were more difficult to be fully infiltrated, then more defects such as voids in the inner part of the agglomerates and gaps in the interface were formed in the nanocomposites, which had been proved to have a positive effect on energy dissipation. In addition, the loss factor of nanocomposites increases with frequency, which is in accordance with the established relationship between the frequency and loss factor [48].

### 3.5. Thermal Stability

**Figure 14:**
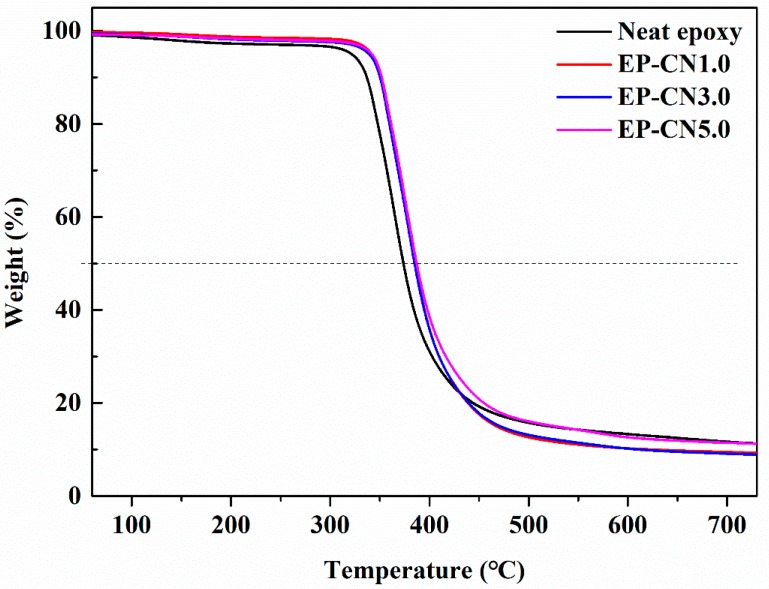
Gravimetric thermograms (TG) of the epoxy and g-C_3_N_4_/epoxy nanocomposites.

The effect of g-C_3_N_4_ on the thermal stability of the epoxy was studied by TGA. The gravimetric thermograms of epoxy and g-C_3_N_4_/epoxy nanocomposites are shown in Figure 14. The 10% weightlessness temperature (T*_initial_*) and semi weightlessness temperature (T*_half_*) are generally regarded as an index of the thermal stability. As displayed in Figure 14, T*_initial_* and T*_half_* of g-C_3_N_4_/epoxy nanocomposites increased by about 15 °C and 14 °C, respectively. This is possibly due to the addition of g-C_3_N_4_ has a hindrance to the decomposition of epoxy resin. Similar results were found in graphene and nano-clay reinforced nanocomposites [49]. The so-called “tortuous path” effect of fillers was considered to be the main reason for the improved thermal stability. The decrease in permeability due to the addition of g-C_3_N_4_ delayed the permeation of oxygen and the escape of volatile degradation products and also char formation [50].

## 4. Conclusions

Here, the g-C_3_N_4_ reinforced epoxy nanocomposites were prepared and characterized. In our experiment, when the loading was less than 3 wt.%, g-C_3_N_4_ can obtain excellent dispersion in epoxy. Results indicated that the static mechanical, dynamic thermal-mechanical and thermal stability properties of g-C_3_N_4_/epoxy nanocomposites had been significantly improved. Compared to neat epoxy, the tensile modulus and flexural modulus of g-C_3_N_4_ reinforced epoxy matrix increased by 31.81% with a loading of 4 wt.% and 28.28% with a loading of 5 wt.%, respectively. However, the maximum values of tensile and flexural strengths were achieved at smaller additions (1–1.5 wt.%). In terms of the magnitude of the enhancement, the enhancement effect of g-C_3_N_4_ on strength was relatively weak. The loss factor of nanocomposites (3 wt.%) under the excitation frequency of 1 Hz at 30 °C increased from 0.035 to 0.049. Meanwhile, the T*_g_* of nanocomposites (1 wt.% and 1.5 wt.%) increased about 8 °C in comparison to that of epoxy matrix and T*_initial_* and T*_half_* increased by about 15 °C and 14 °C, respectively. The structural characteristics of graphite-type carbon nitride, such as multi-scale lamination structure, large specific surface area and naturally carried amino groups, played an excellent role in promoting the dispersion of nanoparticles and strengthening epoxy matrix. The g-C_3_N_4_ as a novel filler for fabricating epoxy nanocomposites is not only feasible but also excellent. The strategy developed in this study could provide a new avenue for the fabrication of high-performance composites. In the follow-up study, we will do more in-depth research on the effect of g-C_3_N_4_ on the friction properties of the epoxy matrix, the effect of the existing form of staking structure in the matrix on the properties of nanocomposites, and the relationship between energy dissipation of hydrogen bond in the interlayer and temperature.
